# Evaluation of Rumen-Protected Methionine Supplementation on Milk Production and Composition in Crossbred Dairy Sheep

**DOI:** 10.3390/ani15070960

**Published:** 2025-03-27

**Authors:** Juan Carlos Angeles-Hernandez, Josué Vicente Cervantes-Bazán, Rodolfo Vieyra-Alberto, Angelica Valeria Lorenzana-Moreno, Ángel Garduño-García, Augusto César Lizarazo-Chaparro

**Affiliations:** 1 Departamento de Medicina y Zootecnia de Rumiantes, Facultad de Medicina Veterinaria y Zootecnia, Universidad Nacional Autónoma de México, Coyoacán, Ciudad de México C.P. 04510, Mexico; juanangeles@fmvz.unam.mx; 2 Departamento de Ingeniería Mecánica Agrícola, Universidad Autónoma Chapingo, Carr. México—Texcoco Km. 38.5, Chapingo, Texcoco C.P. 56230, Estado de México, Mexico; jcervantesba@chapingo.mx (J.V.C.-B.); agardunog@chapingo.mx (Á.G.-G.); 3 Instituto de Ciencias Agropecuarias, Universidad Autónoma del Estado de Hidalgo, Avenida Universidad km 1 s/n, Exhacienda Aquetzalpa, Tulancingo de Bravo C.P. 43600, Hidalgo, Mexico; rodolfo_vieyra@uaeh.edu.mx; 4 Centro de Enseñanza Práctica e Investigación en Producción y Salud Animal, Universidad Nacional Autónoma de México, Avenida Cruz Blanca No. 486, Tlapan, Ciudad de México C.P. 14500, Mexico; mvz.lorenzana@gmail.com

**Keywords:** rumen, milk, protein, dietary supplements, amino acids, rumen-protected methionine, sheep

## Abstract

In dairy sheep, optimum nutrition is required to maintain a positive energy and nitrogen balance, and therefore, achieve adequate performance levels. In this sense, supplemental rumen-protected methionine (RPMet) has been a nutritional strategy used to improve the availability of amino acids in sheep for milk production. The aim of the current study is to assess the effect of RPMet supplementation on the milk yield and milk composition of crossbred dairy sheep. Twenty F1 sheep (50% Pelibuey × 50% East Friesian) were randomly assigned to one of the following three treatments: C (control: 0 g/d), 3 g (3.0 g/d), or 6 g (6 g/d) of RPMet added to the basal diet from day 2 to 120 after lambing. Milk yield was significantly higher in sheep supplemented with 6 g of RPMet (*p* = 0.04) with a linear effect (*p* < 0.001). In relation to protein yield, the 6 g treatment showed the highest values (55.5 g/day) in comparison with the control (35.2 g/day) and 3 g (42.3 g/day) treatments. Also, lactose (*p* = 0.02) and non-fat solid yield (*p* = 0.03) were positively affected by the methionine supplementation. Our results suggest that methionine supplementation is an option to increase milk production, likely associated with the better availability of nutrients of dairy sheep farms using crossbred sheep in regions with an incipient dairy sheep industry.

## Simple Summary

The increasing food demands of the human population determine the need to optimize the animal production process. Dietary supplementation of rumen-protected amino acids has been identified as a useful strategy to increase feed and production efficiency. Dairy sheep production is a novel activity in Latin American countries, providing an opportunity for sheep farmers to increase their incomes. Therefore, the aim of the current work was to evaluate the use of rumen-protected methionine to increase sheep milk production and enhance chemical composition. Our results reveal that supplementation with 6 g/d of rumen-protected methionine improves the milk yield of crossbred sheep. Also, protein, lactose, and non-fat solid yields increased in milk from sheep supplemented with 6 g/d of rumen-protected methionine. For this reason, our results allocate the use of rumen-protected methionine as an option to increase the feed efficiency of dairy sheep production systems in regions with an incipient dairy sheep industry, such as Latin America.

## 1. Introduction

Society’s demand for protein of animal origin is increasing due to the growing global population, especially in developing countries [1]. In order to respond to this trend, ensuring food security and food accessibility, an increase in farming productivity and efficiency is a must. In this sense, since feed accounts for a large proportion of farms’ total cost of production, one of the main strategies to increase the farm’s productivity is to reduce feed intake without decreasing animal production performance [2]. However, the global challenge of increasing food production with even fewer resources leads to the need to improve feed efficiency. In fact, feed efficiency has been identified as one of the main tasks of researchers, farmers, and the feed industry [3]. Moreover, the cost of protein and the environmental effects of nitrogen require special attention [4]. Among the different existing strategies to increase feed and production efficiency, dietary supplementation of rumen-protected amino acids (AAs) has been identified as useful and has become common practice in certain markets and farm profiles in order to meet animals’ requirements of protein for milk production, improve protein and energy efficiency, reduce nutritional costs, and mitigate the environment impact associated with the amount of feed consumed by farm animals [5].

Since the first report of rumen-protected AA supplementation carried out by Broderick et al. [6], who fed dairy cows encapsulated methionine, this AA has been identified as the most limiting AA for lactating dairy cows, goats, and sheep [6]. Methionine is an essential sulfur-containing AA and methyl donor involved in several key physiological processes [7,8]. In lactating animals, the circulating methionine concentrations decrease around parturition, which compromises the milk protein synthesis and utilization of other circulating AAs [9]. Lysine and methionine are likely to be the most limiting amino acids for lactating cows on diets based on corn [10]. Therefore, methionine supplementation in rumen-protected forms is needed since this essential AA is degraded in the rumen [8,11].

Several studies focusing on methionine supplementation have reported increases in milk protein synthesis, milk yield, dry matter intake, reduced oxidative stress, and improved immune function and reproductive performance in dairy animals [7,9,11,12,13]. In sheep, optimal nutrition to maintain a positive energy and nitrogen balance is required to achieve adequate dairy performance. In this sense, supplementation with rumen-protected methionine (RPMet) has also been a nutritional strategy to improve the availability of amino acids for milk production in sheep [14].

In recent decades, Latin American countries have experienced a growing development of the dairy sheep industry as a means of diversifying animal products and increasing economic benefits for sheep producers [15]. Initially, breeds such as East Friesian, Lacaune, and Awassi were introduced to establish specialized sheep milk production systems [16]. However, the challenging agro-climatic conditions of the region have highlighted the high susceptibility of purebred dairy breeds to parasitic and respiratory diseases. Suarez et al. [15]. reported that respiratory diseases are a major challenge for dairy sheep farms in Argentina, with a prevalence of approximately 57.9%. To address these challenges, several studies have evaluated the performance of crossbred dairy sheep, combining specialized dairy breeds with native animals, with the aim of improving adaptation to environmental conditions and enhancing milk yield performance [16,17]. In this context, methionine supplementation has emerged as a promising strategy to optimize the health and productivity of dairy sheep in these unique conditions.

Rumen-protected methionine (RPMet) supplementation in sheep has demonstrated increases in the growth rates of lambs [18], wool growth in mature ewes [19], increases in milk yield, and enhanced feed efficiency for milk production. Goulas et al. [20] mentioned that the positive effect of RPMet supplementation is associated with a better capability of converting dry matter intake into milk, improving the use of AA for milk protein synthesis and optimizing the ratio among essential AAs. Also, Tsiplakou et al. [21] found that supplementation with RPMet has a significant influence on the fatty acid profile in dairy sheep. However, on a practical level, there are several challenges and limitations to evaluating the effects of RPMet supplementation in dairy ruminants. These include difficulties in predicting the ruminal passage of microbial proteins, their post-ruminal absorbability, the amino acid (AA) profile, AA utilization by the gut, and uptake by the mammary gland [22]. Furthermore, some meta-analytic reviews of dairy cows have reported that the positive effects of methionine are limited to improvements in protein and fat yield, with no significant effect on milk yield [8,23]. Regarding the effect of RPMet in dairy sheep, most studies evaluating the effect of RPMet have been developed in specialized dairy breeds from countries with a wide tradition of dairy production. Therefore, the aim of the current study is to address the existing knowledge gap by evaluating the effect of RPMet supplementation on milk yield and composition in crossbred dairy sheep.

## 2. Materials and Methods

### 2.1. Site of Experimentation

The experimental stage of the current study was carried out at the Center of Practice Teaching and Research in Animal Production and Health located at 19°21′ N, 99°05′ W and 2760 m above mean sea level. The experimental site depicts a semi-humid climate with a mean annual temperature of 19 °C and an average annual rainfall of 800 mm. The laboratory analyses were performed in the facilities of the Laboratory of Animal Nutrition of the Faculty of Veterinary Medicine of the National University Autonomous of Mexico.

### 2.2. Animals and Experimental Design

**Figure 1:**
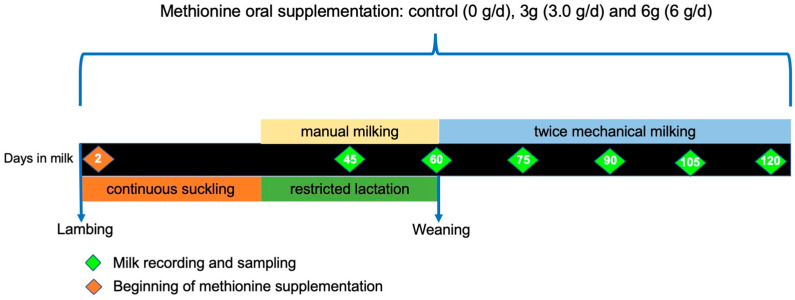
Scheme of experimental procedure for the evaluation of the effect of RPMet supplementation on milk production in crossbred dairy ewes.

An experimental design with repeated measures was used in the current study. Twenty multiparous F1 sheep (50% Pelibuey × 50% East Friesian) with a live weight of 64.0 ± 3.6 kg at the start of the experiment were randomly assigned to one of three treatments. The experimental treatments were control (0 g/d; n = 6), 3.0 g/d (3 g; n = 7), and 6 g/d (6 g; n = 7) of protected methionine (RPMet; Mepron ^®^ Evonik Industries, Essen, Germany) supplemented as a pellet from day 2 to 120 post-lambing using an oral sonde. Sheep milk yield was recorded biweekly, and individual samples were collected for chemical composition analysis up to 120 days post-lambing, with a total of six repeated measures for each animal (Figure 1).

The sample size and power analysis were performed using the G-Power software version 3.1.9.7 [24] with a repeated-measures between-factors design. The inputs that feed G-Power were effect size = 0.33, α = 0.05, power = 0.90, number of groups = 3, number of measures = 6, and correlation among repeated measures = 0.7. The effect size (standardized mean difference) and correlation within subject values were based on the findings of Goulas et al. [20], who tested the effect of methionine on milk sheep production and chemical composition. The power analysis determined that, based on the information provided, a total of 20 animals were required to achieve a power of 0.90 distributed across the three treatments.

### 2.3. Management and Experimental Diets

All procedures of the experiment were approved and observed by the Committee for Care and Use of Experimental Animals (CICUAE-719) of the National Autonomous University of Mexico and were carried out under veterinary care. Lambs suckled their dams freely during the first month of age. Sheep were mechanically milked once daily (08:00 h) from day 31 after lambs were removed during the evening only (from 15:00 h) and milked twice daily after lambs were weaned at 60 days old. The basal diet was formulated to meet the nutrient requirements of lactation sheep based on the National Research Council [25]. The ingredients and composition of the basal diets are shown in Table 1.

### 2.4. Sample Collection and Analytical Procedures

Individual biweekly milk samples were collected in 30 mL plastic tubes and analyzed immediately using an Ekomilk-Standard Milk Analyzer (BULTECH 2000 Ltd., Stara Zagora, Bulgaria) with which the following were determined: protein content, lactose content, fat content, and non-fat solids content. The dry matter, crude protein, ether extract, and crude fiber of feeds were analyzed according to AOAC [26,27] (934.01, 2001.11, 920.39, 942.05, and 962.09, respectively).

### 2.5. Statistical Analysis

A repeated-measures mixed model was implemented to analyze the effect of methionine inclusion on milk yield and chemical composition. Prior to analysis, a Shapiro–Wilk test was performed to assess the normality of the data and a Levene’s test for homoscedasticity. In cases of non-normality, variables were normalized using either logarithmic or exponential transformation. Sheep were uniformly distributed across the three treatments according to their parity and type of lambing. These factors were initially included in the statistical model as blocking effects. In addition, sheep were equally distributed between treatments according to lamb weight, and this factor was included in the statistical model as a covariate. However, as no significant effects were found for these factors, they were excluded from the final statistical model. The final model included the following fixed and random effects:

Y*_ijt_* = µ + Me*_i_* + S*_j_*_(*i*)_ + Time*_t_* + (Me × Time)*_it_* + e*_ijt_*

where:

- Y*_ijt_* is the value of the response variable of the *i*-th level of RPMet of the *j*-th sheep at *t*-th time.
- µ is the overall mean effect.
- Me*_i_* is the fixed effect of the *i*-th level of inclusion of m RPMet (*i* = 0, 3, or 6 g).
- S*_j_*_(*i*)_ is the random effect of the *j*-th sheep within the *i*-th level of inclusion of RPMet.
- Time*_t_* is the fixed *t*-th time effect when the measurement was taken (week of lactation trial).
- (Me × Time)*_it_* is the fixed effect of the interaction between *i*-th level of inclusion of RPMet and *t*-th time.
- e*_ijt_* is the random error.

Several matrices of variances and covariances were tested (autoregressive 1, compound symmetry, and Toeplitz) to choose which depicted the best fitting of our data structure using the Akaike information criterion (AIC) and Bayesian information criterion (BIC) as criteria of goodness of fit [28]. Significance was noted when the *p*-value was lower than 0.05. The mixed models were fitted using the lmer function of the lme4 package [29] of the R statistical program [30]. In the case of rejecting the null hypothesis, post hoc comparisons (Tukey test) were carried out using the emmeans package [31] of the same statistical program.

## 3. Results

### 3.1. Milk Yield Performance

**Figure 2:**
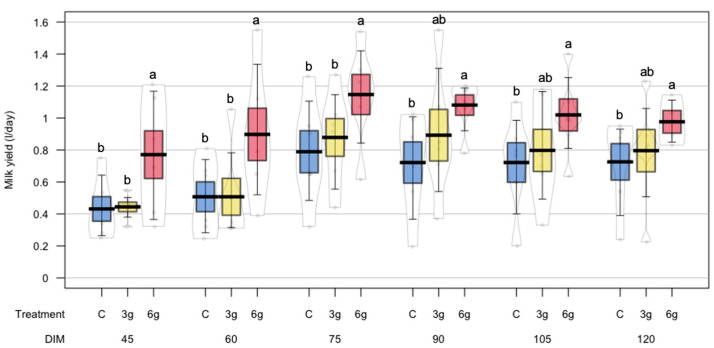
Effect of RPMet supplementation on milk production of crossbred dairy sheep. ^a,b^ Treatment levels in each days in milk (DIM) with different letters are significantly different (*p* < 0.05).

The effect of the inclusion of RPMet on milk yield and milk composition of dairy sheep was evaluated in the current experiment. Descriptive statistics and normality tests are presented in Table 2. Milk yield was significantly higher in sheep supplemented with 6 g of RPMet (*p* = 0.04) with a linear effect (*p* < 0.001) (Table 3). In addition, the 6 g treatment showed a smaller dispersion of their response, especially at 90, 105, and 120 days of lactation (Figure 2).

### 3.2. Milk Composition

**Figure 3:**
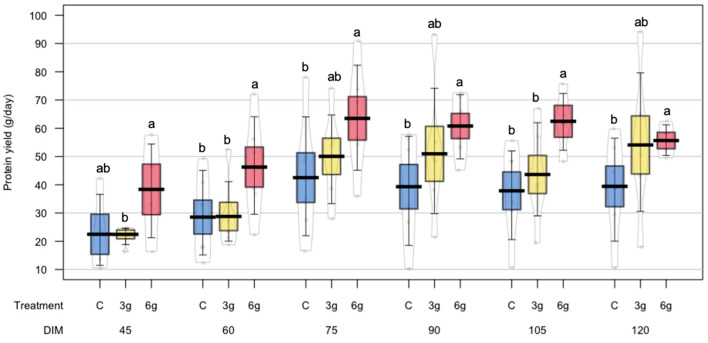
Effect of RPMet supplementation on protein yield (g/day) of sheep milk. ^a,b^ Treatment levels in each days in milk (DIM) with different letters are significantly different (*p* < 0.05).

Protein yield was significantly (*p* = 0.04) affected by RPMet supplementation, with the 6 g treatment showing the highest values compared to the C and 3 g treatments (Table 3). As with milk yield, the 6 g treatment showed a more homogeneous response to methionine supplementation on protein yield, especially at 90, 105, and 120 DIM (Figure 3). A significant linear effect on protein yield was also observed with RPMet supplementation in crossbred dairy ewes. Lactose yield was significantly affected by RPMet supplementation (*p* = 0.02), with differences between the C and 6 g treatments (Table 2). Additionally, a significant linear effect was also observed for this response variable (*p* < 0.001).

**Figure 4:**
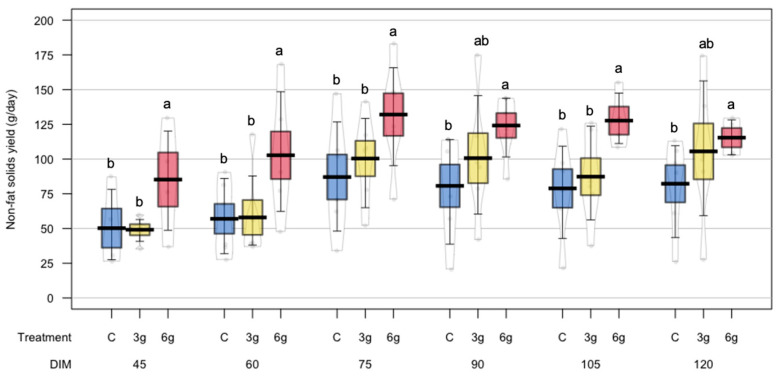
Effect of RPMet supplementation on non-fat solid yields (g/day) of sheep milk. ^a,b^ Treatment levels in each days in milk (DIM) with different letters are significantly different (*p* < 0.05).

The non-fat solid yield showed a significant positive effect (*p* = 0.03) derived from RPMet supplementation, with better values for the 6 g treatment compared to the C animals (Table 3). Figure 4 shows that the 6 g treatment showed the highest values of non-fat solid yield throughout the experiment, with less variability in their response at 90, 105, and 120 days of lactation. On the other hand, total solids content and yield were not affected by the inclusion of RPMet in dairy ewes’ rations (*p* > 0.05) (Table 3). In the same sense, no significant differences were observed as an effect of RPMet supplementation on fat content (*p* = 0.43) and fat yield (*p* = 0.41) (Table 3). A significant quadratic effect was observed on protein content (*p* < 0.001); however, the effect of RPMet supplementation and the interaction of Met × time were not significant (*p* > 0.05).

## 4. Discussion

### 4.1. Milk Yield Performance

The observed increase in milk yield of crossbred sheep supplemented with RPMet could be related to the enhancement of the availability of nutrients due to the positive effect on dry matter intake (DMI) reported by methionine supplementation. Lopreitano et al. [11] mentioned that methionine supplementation helps to maintain a constant DMI during prepartum and promotes an increase in DMI in early lactation. Also, Goulas et al. [20] concluded that the better milk yield performance of sheep supplemented with RPMet is due to the increased absorption of limiting nutrients of the diet, improving the protein balance and indicating higher availability of AA. On the other hand, our results contrast with other studies that did not show a positive response on milk yield when RPMet was supplemented in dairy sheep and goats [14,32], which can be attributed to the fact that methionine was not limiting AA in these studies [21] or the capability of ruminants to maintain a supply of AA to the mammary gland to support milk production regardless of the AA profile of fed diets [33].

### 4.2. Milk Composition

RPMet supplementation had no effect on milk fat yield and fat content in the current trial, in agreement with the results of Wiltbank et al. [13]. However, our findings contrast with other studies that reported an increase in milk fat content derived from RPMet supplementation in sheep [5,34,35], which could be explained, in part, by the role of methionine as a methyl group supplier, choline synthesis [36], and lipid transportation [37]. In the meta-analysis developed by Zanton et al. [8], milk fat yield and fat concentration were significantly affected by the methionine supplementation, but they also concluded that the level of response depends on the sources of methionine. Hence, between-study variability can be explained by factors such as the source of methionine, level of supplementation, components used to protect methionine, imbalance of other Aas, and characteristics of feed rations in each experiment [8,12].

This was likely associated with a dilution effect because there was an increase in milk yield; however, the RPMet effect was evident in milk protein yield. The increase in milk protein yield indicates that the basal diet does not contain enough methionine [38]; therefore, RPMet fed to ruminants promoted greater circulating plasma levels of met [39] and some other essential AAs (i.e., Arg, Lys, Thr, and Trp), non-essential AAs (Ala, Asn, Asp, Glu, Gln, and pro) and non proteininc AAs (Cit) for milk protein synthesis [7]. Also, milk protein is composed of 80% casein (~30% ß-casein), whereby the higher milk protein yield of RPMet-supplemented sheep could be partially explained by the fact that methionine increases the ß-casein expression in the mammary gland [40]. This is supported by previous studies that reported an increase in milk protein as a result of casein abomasal infusions [41,42].

In this study, the dietary supplementation of RPMet in crossbred sheep did not affect lactose content, which is in agreement with previous studies on sheep [5,21] and goats [33]. However, our results are in accordance with studies that reported a significant effect of dietary RPMet supplementation on lactose yield in early sheep lactations [20]. This effect can be attributed to the increase in milk yield and the role of lactose as a major osmotic component determining milk yield via the water uptake in the secretion vesicles of lactocytes [43], whereby higher milk production is derived from a proportional increase in lactose synthesis by the mammary gland [44,45].

## 5. Conclusions

The results of the current study support the previous findings of the positive effect of dietary RPMet supplementation on milk yield and composition in sheep. Moreover, the level of response is dependent on the supplementation dosage, with better effects on milk yield, protein yield, and lactose yield with a dosage of 6 g/day. Our results indicate that methionine supplementation is an option to increase milk production, likely associated with the better availability of nutrients of dairy sheep farms using crossbred sheep in regions with an incipient dairy sheep industry.

## Figures and Tables

**Table 1:** Ingredients and composition of the basal diet (% of DM) provided to crossbred dairy sheep.

Item	Alfalfa Pellet	Corn Silage	Oat Hay	Commercial Concentrate	Mineral Premix ^1^	Basal Diet
% Inclusion	23.0	15.0	35.0	25.0	2.0	100.0
Dry matter (g/kg)	960.0	232.0	924.0	930.0	100.0	81.31
Crude protein (g/kg)	188.41	67.45	49.83	194.0	0	105.15
Ether extract (g/kg)	9.81	3.22	11.44	37.31	0	146.34
NDF (g/kg)	420.0	648.23	642.0	387.53	0	413.01
ADF (g/kg)	300.0	405.21	433.39	251.87	0	279.04
Ca (g/kg)	1.41	0.31	0.32	1.81	140.0	1.12
P (g/kg)	0.22	0.27	0.25	0.62	65.0	0.41

NDF, neutral detergent fiber; ADF, acid detergent fiber. ^1^ Provided per kilogram of premix: Mn 6000 mg, Fe 4000 mg, Cu 3000 mg, 1260 mg, Se 20 mg, vitamin A 500,000 IU, vitamin D 85,000 IU, vitamin E 5000 IU.

**Table 2:** Descriptive statistics and test of normality of milk production and composition variables.

Item	Mean	SD	Min	Max	Skewness	Kurtosis	*p* Value (Shapiro-Wilk)
Milk yield (L/day)	0.78	0.34	0.2	1.55	0.1	−0.79	0.29
Fat content (g/100 g)	5.34	2.19	2.8	12.5	0.03	0.28	0.06
Fat yield (g/day)	43.4	24.96	10.17	111.14	0.14	−0.70	0.14
Protein content (g/100 g)	5.48	0.63	4.22	7.97	0.23	0.55	0.07
Protein yield (g/day)	43.86	19.74	10.22	94.22	0.24	−0.48	0.05
Lactose content (g/100 g)	4.79	0.35	3.39	5.94	0.69	2.5	0.004
Lactose yield (g/day)	38.45	16.84	20.71	80.44	0.04	−0.79	0.08
Total solid content (g/100 g)	15.36	2.37	10.02	23.67	0.35	0.85	0.02
Total solid yield (g/day)	124.08	57.60	27.90	268.92	0.08	−0.81	0.06
Non-fat solids content (g/100 g)	11.29	0.51	95.70	125.7	0.08	0.35	0.66
Non-fat solids yield (g/day)	90.31	39.45	20.51	183.11	0.10	−0.76	0.07

**Table 3:** Effect of level RPMet supplementation on milk production and composition of crossbred dairy sheep.

Item	Level of Methionine				*p* Value			Effect	
	C	3 g	6 g	SEM	Met	Time	Met × Time	L	Q
Lamb birth weight (kg)	4.11	4.33	4.19	0.16	0.87			ns	ns
Milk yield (L/day)	0.65 ^b^	0.72 ^b^	0.98 ^a^	0.08	0.04	0.0001	0.97	**	ns
Fat content (g/100 g)	5.53	5.52	4.93	0.43	0.43	0.0001	0.17	ns	ns
Fat yield (g/day)	38.21	42.12	51.93	6.39	0.41	0.0001	0.25	ns	ns
Protein content (g/100 g)	5.22	5.69	5.25	0.20	0.33	0.0001	0.26	ns	***
Protein yield (g/day)	35.23 ^b^	42.29 ^b^	55.51 ^a^	5.31	0.04	0.0001	0.42	***	ns
Lactose content (g/100 g)	4.74	4.65	5.04	0.09	0.54	0.0001	0.30	ns	***
Lactose yield (g/day)	31.34 ^b^	35.02 ^ab^	50.88 ^a^	6.38	0.02	0.0002	0.57	***	ns
Total solid content (g/100 g)	15.04	15.878	15.32	0.46	0.71	0.03	0.53	ns	ns
Total solid yield (g/day)	10.21	11.86	15.73	1.51	0.07	0.0001	0.33	***	ns
Non-fat solids content (g/100 g)	11.03	11.41	11.53	0.23	0.18	0.04	0.56	ns	ns
Non-fat solids yield (g/day)	73.02 ^b^	85.04 ^ab^	116.04 ^a^	1.49	0.03	0.0001	0.51	**	ns

** *p* < 0.01; *** *p* < 0.001; ^a,b^ Values within rows followed by different letters are significantly different (*p* < 0.05); C, control (without methionine); Met, methionine effect; ns, no significance; L, linear effect; Q, quadratic effect.

## Data Availability

Data are contained within the article.
